# Crowd-sourced and expert video assessment in minimally invasive esophagectomy

**DOI:** 10.1007/s00464-023-10297-2

**Published:** 2023-08-21

**Authors:** Mirte H. M. Ketel, Bastiaan R. Klarenbeek, Yassin Eddahchouri, Miguel A. Cuesta, Elke van Daele, Christian A. Gutschow, Arnulf H. Hölscher, Michal Hubka, Misha D. P. Luyer, Robert E. Merritt, Grard A. P. Nieuwenhuijzen, Yaxing Shen, Inger L. Abma, Camiel Rosman, Frans van Workum

**Affiliations:** 1 Department of Surgery, Radboud University Medical Center, Nijmegen, The Netherlands; 2 Department of Surgery, Amsterdam University Medical Centers, Location VUmc, Amsterdam, The Netherlands; 3 Department of Digestive Surgery, Ghent University Hospital, Ghent, Belgium; 4 Department of Surgery and Transplantation, University Hospital Zurich, Zurich, Switzerland; 5 Department for General, Visceral and Trauma Surgery, Elisabeth-Krankenhaus-Essen GmbH, Essen, Germany; 6 Department of Thoracic Surgery, Virginia Mason Medical Center, Seattle, SE USA; 7 Department of Surgery, Catharina Hospital, Eindhoven, The Netherlands; 8 Department of Surgery, Ohio State University - Wexner Medical Center, Columbus, OH USA; 9 Department of Thoracic Surgery, Zhongshan Hospital, Fudan University, Shanghai, China; 10 IQ Healthcare, Radboud University Medical Center, Nijmegen, The Netherlands; 11 Department of Surgery, Canisius-Wilhelmina Hospital, Nijmegen, The Netherlands

**Keywords:** Crowdsourcing, GOALS, Competency assessment tool, Surgical performance assessment, Esophagectomy, Video assessment

## Abstract

**Background:**

Video-based assessment by experts may structurally measure surgical performance using procedure-specific competency assessment tools (CATs). A CAT for minimally invasive esophagectomy (MIE-CAT) was developed and validated previously. However, surgeon’s time is scarce and video assessment is time-consuming and labor intensive. This study investigated non-procedure-specific assessment of MIE video clips by MIE experts and crowdsourcing, collective surgical performance evaluation by anonymous and untrained laypeople, to assist procedure-specific expert review.

**Methods:**

Two surgical performance scoring frameworks were used to assess eight MIE videos. First, global performance was assessed with the non-procedure-specific Global Operative Assessment of Laparoscopic Skills (GOALS) of 64 procedural phase-based video clips < 10 min. Each clip was assessed by two MIE experts and > 30 crowd workers. Second, the same experts assessed procedure-specific performance with the MIE-CAT of the corresponding full-length video. Reliability and convergent validity of GOALS for MIE were investigated using hypothesis testing with correlations (experience, blood loss, operative time, and MIE-CAT).

**Results:**

Less than 75% of hypothesized correlations between GOALS scores and experience of the surgical team (*r *< 0.3), blood loss (*r* = − 0.82 to 0.02), operative time (*r* = − 0.42 to 0.07), and the MIE-CAT scores (*r* = − 0.04 to 0.76) were met for both crowd workers and experts. Interestingly, experts’ GOALS and MIE-CAT scores correlated strongly (*r* = 0.40 to 0.79), while crowd workers’ GOALS and experts’ MIE-CAT scores correlations were weak (*r* = − 0.04 to 0.49). Expert and crowd worker GOALS scores correlated poorly (ICC ≤ 0.42).

**Conclusion:**

GOALS assessments by crowd workers lacked convergent validity and showed poor reliability. It is likely that MIE is technically too difficult to assess for laypeople. Convergent validity of GOALS assessments by experts could also not be established. GOALS might not be comprehensive enough to assess detailed MIE performance. However, expert’s GOALS and MIE-CAT scores strongly correlated indicating video clip (instead of full-length video) assessments could be useful to shorten assessment time.

**Graphical abstract:**

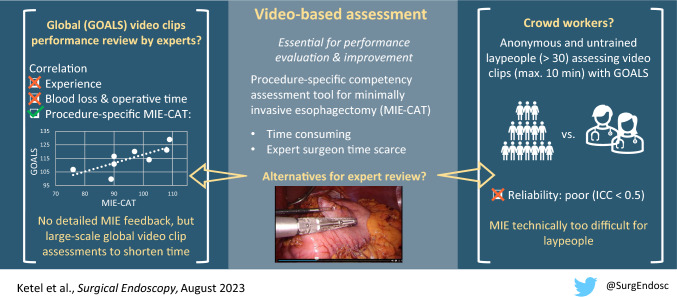

**Supplementary Information:**

The online version contains supplementary material available at 10.1007/s00464-023-10297-2.

Suboptimal surgical performance has been related to less favorable patient outcomes in complex minimally invasive surgical procedures [1–5]. Procedure-specific competency assessment tools (CATs) are an objective method to measure surgical performance with [2, 6, 7]. CATs are tools for structured (video-based) assessment used by experts to assess surgical performance and are currently viewed as the gold standard for measuring surgical performance for a given surgical procedure. A procedure-specific CAT for minimally invasive esophagectomy (MIE), the MIE-CAT, was developed and validated by our group previously [8]. However, an important limitation for using the MIE-CAT is that time of expert surgeons is scarce and video analysis is time-consuming and labor intensive, thereby limiting potential broad applicability [2].

A way to assist expert review for MIE might be using global performance assessment of video clips. Global performance assessment with Global Operative Assessment of Laparoscopic Skills (GOALS) [9] could provide an initial performance estimate, potentially followed by detailed feedback of the MIE-CAT if desired. In addition, using video clips instead of full-length videos might reduce assessment time without loss of relevant information, but could introduce bias [10]. Another way to assist expert review might be the use of crowdsourcing, in which large groups of anonymous and untrained workers evaluate surgical performance [11–15]. However, previous studies that investigated crowdsourcing for surgical performance video assessment have important limitations: (1) the outcomes of non-procedure-specific tools (e.g., GOALS or Global Evaluative Assessment of Robotic Skills (GEARS) [16]) were not compared to procedure-specific CATs; and (2) the studies were performed with videos of relatively simple tasks or procedures, often in dry-lab settings [15, 17–22]. Therefore, additional research is desired to explore both global performance assessment of video clips and crowdsourcing for video assessment of MIE.

There are two primary aims of this study: (1) to evaluate global performance assessment of MIE and (2) to evaluate the correlation between MIE experts and crowd workers assessments. We hypothesize that global performance assessment by both MIE experts and crowd workers can reliably be used to assist expert review, by providing rapid global feedback for MIE surgeons and reduce assessment time significantly.

## Materials and methods

**Fig. 1:**
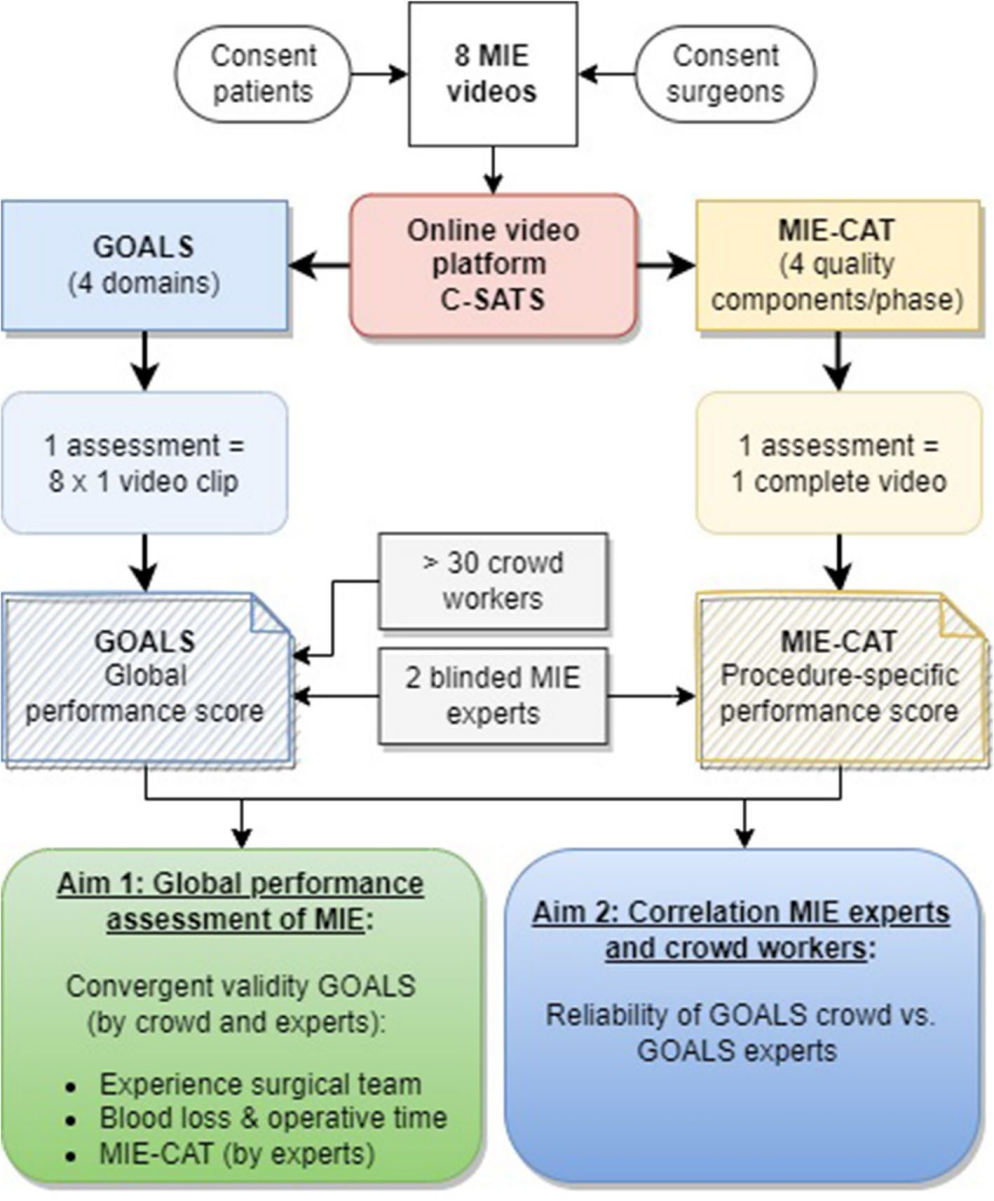
Overview of the study with two types of video assessments: left GOALS and on the right MIE-CAT assessments

Two frameworks for scoring surgical performance of MIE were used in this study: (1) global performance assessment (GOALS) and (2) procedure-specific performance (MIE-CAT) (Fig. 1). GOALS was assessed by both crowd workers and MIE experts, while the MIE-CAT was only assessed by MIE experts. The use of the MIE-CAT for crowd workers was considered unfeasible given the technical complexity of the MIE-CAT. Reliability and convergent validity of the surgical performance assessments were analyzed as constructed by the COSMIN panel [23]. This study was carried out in accordance with applicable legislation and reviewed by the ethical committee of the Radboud university medical center.

### Video database and pre-processing

Full-length intraoperative MIE videos, performed thoraco-laparoscopically between 2011 and 2020, from the database of the Esophageal Center East Netherlands in Nijmegen were used. Eight transthoracic MIE with intrathoracic anastomosis videos for esophageal cancer were randomly included. Each included procedure was performed by two consultant surgeons from one surgical team with four consultant surgeons in total. Procedures assisted by surgeons in training were excluded. Videos were divided into four experience groups, based on the consecutive case date and the learning curve of 119 cases found by van Workum et al. [24]. These four groups include (1) novice (0 to 25 MIEs performed), (2) intermediate (26 to 119 MIEs performed), (3) advanced (120 to 200 MIEs performed), and (4) expert (> 200 MIEs performed) (Online Appendix A). Two randomly selected videos per experience group were included after written informed patient consent and were stripped from any patient or surgeon identifiers.

Video clips of maximum 10 min were used for the global performance assessment with GOALS. Every video was cut into eight video clips, with every clip representative to one of the eight phases of the MIE-CAT [8] (Table 1) based on procedural-phase landmarks (Online Appendix A), resulting in 64 video clips total. The eight full-length MIE videos (average 3.5 h per video) were used for the procedure-specific assessments with the MIE-CAT.

**Table 1:** Description of the eight phases of the MIE procedure from the MIE-CAT [8]

Phase	Description
1	Mobilization of the greater curvature
2	Mobilization of the lesser curvature
3	Dissection of the abdominal lymph nodes
4	Dissection of the hiatus
5	Creation of the gastric tube
6	Mobilization of the thoracic esophagus
7	Dissection of the thoracic lymph nodes
8	Creation of the intrathoracic anastomosis

### Reviewers

Crowd workers were recruited by Crowd-Sourced Assessment of Technical Skills® (C-SATS, Seattle, WA, USA) via Amazon Mechanical Turk (MTurk, https://mturk.com), an online marketplace that facilitates hiring crowd workers [25]. Crowd workers were eligible if they were 18 years or older, had more than 95% approval on previous evaluations and lived in the USA. All crowd workers received a training video prior to the assessment, including a skill comparison test with dry-lab videos of a high- and low-performing surgeon. Crowd workers were invited on a ‘first come, first served’ basis.

MIE experts, identified through a literature search in the field of MIE and previous research collaborations [8, 26–28], were contacted to participate in this study. In total, 9 MIE experts who performed at least 120 MIEs [24] and currently perform at least 50 MIEs annually from Europe (6), the USA (2), and Asia (1) were included. The average experience in esophageal surgery was 18 years (SD 8.3). All experts were trained in online video assessment using the MIE-CAT [8] during interactive online workshops of 1.5 h with two to five experts.

### Performance assessment tools

All video assessments were conducted via the online C-SATS platform. First, global performance assessment was conducted by both crowd workers and experts with the non-procedure-specific validated GOALS tool [9] of the 64 video clips. Intraoperative laparoscopic performance of every video clip was assessed using four domains: (1) depth perception, (2) bimanual dexterity, (3) efficiency, and (4) tissue handling. GOALS’ autonomy domain was excluded because of the absence of sound. Each domain was scored with a 1–5 Likert scale. One GOALS assessment included all eight procedural phase-based video clips from one MIE procedure. To obtain a 95% confidence interval to be ± 1 point on the grading scale, at least 30 crowd workers per video clip assessment were deemed necessary based on previous research [17, 18, 29, 30]. In addition, two MIE experts were randomly appointed per GOALS assessment. An average GOALS score for each surgical phase video clip and for the full-length videos were analyzed. The average GOALS score was calculated as mean of all crowd workers or expert assessments.

Second, procedure-specific performance assessment of the eight corresponding full-length MIE videos were assessed with the MIE-CAT by the same two MIE experts. Performance was assessed using the eight procedural phases of the MIE-CAT (Table 1) [8]. Each procedural phase was scored with four quality components (exposure, execution, adverse events, and end-product quality) on a 1–4 Likert scale [8]. Crowd workers were excluded from procedure-specific assessments, since these assessments require procedure-specific expertise. Procedure-specific assessments resulted in one average MIE-CAT score per phase and per full-length video.

### Global performance assessment of MIE

Convergent validity verifies whether the GOALS scores of MIE performance correlates with similar constructs to the degree that one would expect via hypotheses testing [31, 32]. If at least 75% of the hypotheses are correct, convergent validity is considered to be sufficient for GOALS assessment of MIE performance [31]. GOALS components scores that were investigated included the domain scores (4), phase scores (1), and total GOALS score (1) per video. For hypothesis testing the following related construct correlations with GOALS (components) were studied using Pearson’s correlations coefficients:

1. Experience: Correlations between the GOALS (components) scores and experience of the surgical team, defined by consecutive case date. With the six GOALS components in total six hypotheses were tested for each group (crowd workers and experts separately).
2. Clinical parameters: Correlations between the GOALS (components) scores and two clinical parameters: blood loss (in milliliters) and operative time (in minutes). With the six GOALS components in total 12 hypotheses (6 × 2 hypotheses) were tested for each group (crowd workers and experts separately).
3. Procedure-specific performance: Correlations between the GOALS (components) scores, assessed by crowd workers and experts, and the MIE-CAT (components) scores assessed by experts. Components scores were investigated in relation each other: GOALS domain versus MIE-CAT quality component (4), GOALS phase versus MIE-CAT phase (1), and total GOALS versus total MIE-CAT (1). Therefore, in total six hypotheses were tested for each group (crowd workers and experts separately).

First, positive correlations between 0.3 and 0.7 (‘moderate’) were considered acceptable for the hypothesized correlations between GOALS and experience [32]. Second, negative correlations between − 0.3 and − 0.7 (‘moderate’) were considered acceptable for the hypothesized correlations between GOALS and the two clinical parameters. Both the experience of the surgical team and the two clinical parameters were expected to be indicators for global performance of MIE, and this was also confirmed in our previous study for procedure-specific performance for MIE [8]. In addition, Vassiliou et al. [9] showed increased GOALS score with increased experience for laparoscopic cholecystectomy. Smaller correlations than (−)0.3 would indicate inadequate global performance assessment and a higher correlation than (−)0.7 would indicate GOALS only correlates with experience or clinical outcome, while it is expected that global surgical performance embodies both. Third, a strong but not perfect positive correlation between 0.5 and 0.8 between global performance assessment (GOALS) and procedure-specific performance assessment (MIE-CAT) was considered acceptable. Both scoring frameworks are expected to assess surgical performance of MIE and therefore correlate at least moderately, > 0.5. However, the GOALS scores were not expected to correlate more than strong, > 0.8. The MIE-CAT assesses more detailed performance and thus the two tools assess the construct of quality performance in different ways. See Online Appendix B for an overview of the hypotheses for convergent validity. Reliability and validity of the MIE-CAT were established in a previous study [8].

### Correlation MIE experts and crowd workers

The average GOALS scores of both experts and the average GOALS scores of all crowd workers from the corresponding video clip were used to determine inter-rater reliability. This inter-rater reliability was calculated using a one-way intraclass correlation coefficient (ICC) with a mean rating and consistency agreement. An ICC above 0.7 was considered acceptable and ≥ 0.8 good. In addition, a rank order of the lowest to highest scoring video (clip) and Bland–Altman plot was made to display the inter-rater agreement. The data analysis was performed using IBM SPSS Statistics for Windows version 27.0 (IBM Corp., Orchard Road Armonk, New York, US).

## Results

### MIE videos

Patients from the eight randomly included videos had a median age of 68.5 years (IQR 55.5–70.5) and BMI of 25.6 (IQR 21.3–29.2). A detailed overview can be found in Online Appendix C.

### Assessments

The nine experts conducted a total of 16 full-length video assessments (containing ~ 51 video hours) with the MIE-CAT and 128 video clip assessments (containing ~ 21 video hours) with GOALS in 3.5 months. Crowd workers assessed a total of 1984 video clips (containing over 330 video hours) with GOALS in less than 2 days. Overall, the experts and crowd workers showed comparable mean GOALS scores (Table 2). Interestingly, the GOALS video clips scores from experts had a wider range than those of crowd workers (7.50–19.50 versus 12.84–15.63). See Online Appendix D for an overview of the correlating MIE-CAT scores.

**Table 2:** Mean domain, video clip, and total video scores assessed with GOALS by both experts and crowd workers (*n* = 64 video clips)

GOALS component	Experts [range]	Crowd workers [range]	ICC experts crowd (95% CI)
Depth perception (*n* = 64)	3.65 [1.50–5.00]	3.40 [2.81–3.81]	0.09 [− 0.49, 0.49]
Bimanual dexterity (*n* = 64)	3.57 [2.25–5.00]	3.64 [3.19–4.00]	0.23 [− 0.26, 0.53]
Efficiency (*n* = 64)	3.55 [1.00–5.00]	3.60 [3.13–4.03]	0.19 [− 0.33, 0.51]
Tissue handling (*n* = 64)	3.58 [2.00–5.00]	3.70 [3.19–4.25]	− 0.08 [− 0.77, 0.34]
GOALS phase score (*n* = 64)	14.34 [7.50–19.50]	14.34 [12.84–15.63]	0.18 [− 0.35, 0.50]
Total GOALS score (*n* = 8)	114.75 [99.75–128.92]	114.64 [110.83–117.54]	0.42 [− 1.63, 0.88]

In addition, the ICC with 95% CI between experts and crowd workers is shown

### Global performance assessment of MIE

Less than 75% of the correlations between GOALS component scores and the related constructs experience (Table 3), clinical parameters (Table 3), and MIE-CAT components (Table 4) were in agreement with our hypotheses for both the crowd and experts GOALS scores. For crowd workers, 14% of the correlations (5 of 36 hypotheses) and for experts, 56% of the correlations (20 of 36 hypotheses) were in agreement with our hypotheses.

**Table 3:** Correlations between GOALS components (domain, phase, and total scores) by both crowd workers and experts and the related constructs experience and the two clinical parameters

	Domain ‘Depth perception’	Domain ‘Bimanual dexterity’	Domain ‘Efficiency’	Domain ‘Tissue handling’	Phase	Total GOALS
Experience						
Crowd workers	0.10 (− 0.15, 0.34)	0.01 (− 0.23, 0.26)	0.13 (− 0.12, 0.37)	0.14 (− 0.11, 0.37)	0.14 (− 0.11, 0.38)	0.29 (− 0.11, 0.38)
Experts	− 0.07 (− 0.31, 0.18)	− 0.02 (− 0.26, 0.23)	0.05 (− 0.20, 0.29)	− 0.10 (− 0.34, 0.15)	0.04 (− 0.28, 0.21)	− 0.09 (− 0.52, 0.83)
Blood loss						
Crowd workers	− 0.13 (− 0.80, 0.69)	− **0.66 **(− **0.95**, **0.18**)	− **0.54 **(− **0.92**, **0.36**)	0.02 (− 0.75, 76)	− **0.39 **(− **0.88**, **0.52**)	− **0.39 **(− **0.88**, **0.52**)
Experts	− 0.82 (− 0.97,− 0.16)^a^	− **0.62 **(− **0.94**, **0.25**)	− 0.82 (− 0.97,− 0.16)^a^	− **0.46 **(− **0.90**, **0.45**)	− 0.81 (− 0.97,− 0.14)^a^	− 0.81 (− 0.97,− 0.14)^a^
Operative time						
Crowd workers	0.07 (− 0.67, 0.74)	− 0.10 (− 0.75, 0.65)	− **0.34 **(− **84**, **0.48**)	− 0.21 (− 0.80, 0.58)	− 0.15 (− 0.77, 0.62)	− 0.15 (− 0.77, 0.62)
Experts	− 0.14 (− 0.77, 0.63)	− **0.33 **(− **84**, **0.49**)	− **0.42 **(− **87**, **0.42**)	0.12 (− 64, 0.76)	− 0.20 (− 79, 59)	− 0.19 (− 0.79, 0.59)

All bold correlations are within (−)0.3 and (−)0.7

^a^Correlations were found to have a *p* value < 0.05

**Table 4:** Correlations between GOALS components (domain, phase, and total scores) by both crowd workers and experts and the related construct MIE-CAT components (quality components, phase, and total scores) by experts

MIE-CAT	GOALS					
	Domain ‘Depth perception’	Domain ‘Bimanual dexterity’	Domain ‘Efficiency’	Domain ‘Tissue handling’	Phase score	Total GOALS score
Exposure						
Crowd workers	− 0.04 (− 0.72, 0.69)	0.49 (− 0.32, 0.89)	0.11 (− 0.64, 0.76)	0.19 (− 0.60, 0.79)		
Experts	**0.75 (0.09, 0.95)**^a^	**0.79 (0.20, 0.96)**^a^	*0.68 (− 0.04, 0.94)*	**0.53 (− 0.28, 0.90)**		
Execution						
Crowd workers	− 0.04 (0.72, 0.69)	0.34 (− 0.48, 0.84)	0.09 (− 0.66, 0.75)	0.08 (− 0.66, 0.75)		
Experts	**0.77 (0.15, 0.96)**^a^	**0.77 (0.13, 0.96)**^a^	**0.61 (− 0.16, 0.92)**	**0.59 (− 0.19, 0.92)**		
Adverse events						
Crowd workers	0.17 (− 0.61, 0.78)	0.33 (− 0.49, 0.84)	0.28 (− 0.53, 0.82)	0.14 (− 0.62, 0.77)		
Experts	**0.73 (0.05, 0.95)**^a^	**0.63 (− 0.13, 0.93)**	**0.56 (− 0.23, 0.91)**	**0.61 (− 0.17, 0.92)**		
End-product quality						
Crowd workers	0.08 (− 0.66, 0.74)	0.37 (− 0.46, 0.85)	− 0.19 (− 0.79, 0.59)	0.23 (− 0.57, 0.81)		
Experts	**0.65 (− 0.11, 0.93)**	**0.56 (− 0.25, 0.91)**	0.40 (− 0.42, 0.86)	**0.71 (0.02, 0.94)**^a^		
Phase score						
Crowd workers					0.02 (− 0.23, 0.26)	
Experts					0.45 (0.23, 0.63)^a^	
Total MIE-CAT score						
Crowd workers						0.19 (− 0.59, 0.79)
Experts						**0.76 (0.12, 0.95)**^a^

All bold correlations are within 0.5 and 0.8

^a^Correlations were found to have a *p* value < 0.05

**Fig. 2:**
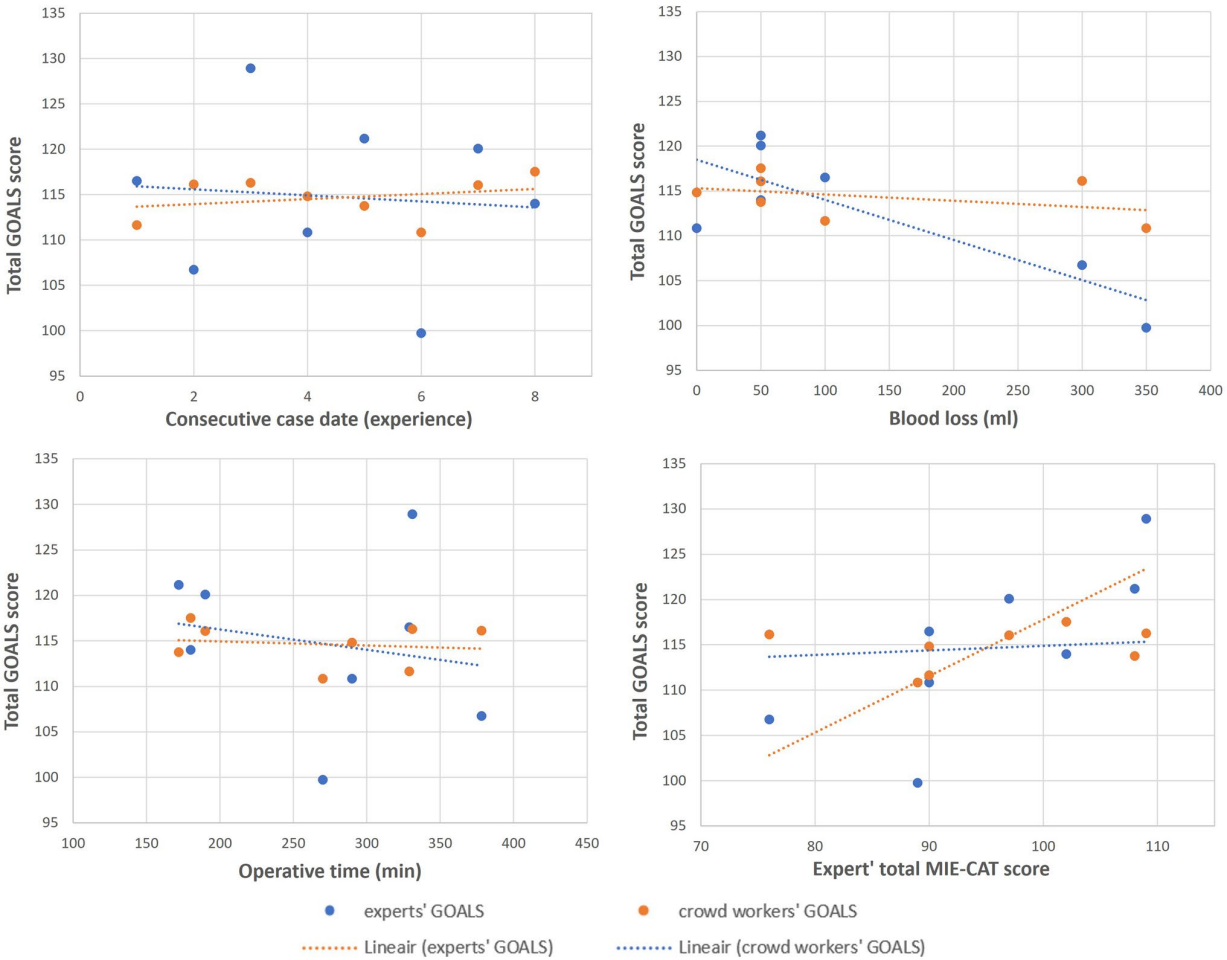
Total GOALS video clip scores assessed by experts (blue) and crowd workers (orange) versus **a** experience of the surgical team in consecutive case date, **b** blood loss (ml) **c** operative time (min), and **d** total MIE-CAT score assessed by experts, all with a linear fitted line (Color figure online)

There was a poor correlation (*r* < 0.3) between the GOALS scores and experience of the surgical team for either group (Table 3, Fig. 2a). None of these correlations (0/12) fell within the hypothesized values of 0.3 < *r* < 0.7. Between the GOALS scores and the two clinical parameters (blood loss & operative time), some correlations did fall within the hypothesized values (Table 3, Fig. 2b and c). For crowd workers, 5/12 correlations (blood loss versus ‘bimanual dexterity,’ ‘efficiency,’ ‘phase,’ and ‘total’ GOALS scores and ‘efficiency’ versus operative time) and for experts 4/12 correlations (blood loss versus ‘bimanual dexterity’/‘tissue handling’ and operative time versus ‘bimanual dexterity’/’efficiency’) were between -0.3 and -0.7, as hypothesized. The correlations between blood loss and ‘bimanual dexterity,’ and the correlation between operative time and ‘efficiency’, were within hypothesized values for both groups. Correlations between the expert GOALS scores and blood loss outside the hypothesized values were all stronger than expected (*r* > − 0.8).

Almost all correlations (16/18) between the experts’ GOALS scores and the MIE-CAT scores were between the hypothesized values of 0.5 and 0.8 (Table 4). The two correlations that correlated less strongly than expected (< 0.5) were between GOALS domain ‘efficiency’ and MIE-CAT quality component ‘end-product quality’ (*r* = 0.40, 95% CI [− 0.42, 0.86]) and between GOALS ‘phase’ and MIE-CAT ‘phase’ scores (*r* = 0.45, 95% CI [0.23, 0.63]). None of the correlations between crowd workers’ GOALS scores and the MIE-CAT were above 0.5 (Table 4). Figure 2d visualizes the strong correlation between the experts’ total GOALS scores and the expert’ total MIE-CAT scores (*r* = 0.76, 95% CI [0.12, 0.95]) versus the minimal correlation between the crowd workers’ GOALS and expert MIE-CAT scores (*r* = 0.19, 95% CI [− 0.59, 0.79]).

### Correlation MIE experts and crowd workers

A poor (ICC < 0.5) level of agreement on domain, video clip, and total GOALS scores was found between the experts and crowd workers (Table 2). All ICC values were below the acceptable 0.7, indicating insufficient agreement. In addition, a proportional bias can be seen (Online Appendix E): experts more frequently scored substantially lower or higher than average, while crowd workers scored the same video clip as ‘average.’ Additionally, only the lowest scoring video was agreed upon by both groups, when ranking the videos based on their total GOALS score (Online Appendix E).

## Discussion

In this study, crowd workers and MIE experts scored 64 video clips of MIE using GOALS, which were compared to each other and to eight full-length video assessments using the MIE-CAT. The first aim was to evaluate global performance assessment with GOALS for MIE using convergent validity. We were unable to establish convergent validity for GOALS, whether scored by crowd workers or experts. However, GOALS scored by experts (but not crowd workers) strongly correlated with the experts’ MIE-CAT scores. This suggests video clip assessment by experts using GOALS could be useful to shorten assessment time when in-depth video analysis using the MIE-CAT is not required. The second aim was to evaluate the correlation between MIE experts and crowd workers assessments. A poor correlation between the crowd workers and experts’ GOALS scores was observed, despite promising results in previous studies and rapid results from crowd workers.

This study found a poor correlation between GOALS scores of crowd workers and experts, in contrast to earlier findings [13–15]. Previous studies, with a comparable sample size of crowd workers, found that dry-lab or relatively simple laparoscopic task video assessments show moderate to good agreement between crowd workers and experts [15, 17–21], while low agreement is seen in more complex laparoscopic global performance assessment videos [30]. Crowd workers might be used for relative simple procedures, whereas findings of the current study suggest performance assessment of real life surgeries, especially complex procedures, such as MIE, might technically be too difficult for laypeople to assess [18].

Although we were unable to establish convergent validity for GOALS, whether scored by crowds or experts, GOALS scored by experts (but not crowd workers) strongly correlated with the experts’ MIE-CAT scores [8]. In our view, GOALS is not comprehensive enough to fully capture the quality of a specific complex surgery, such as MIE. This could explain the poor correlation with experience, leading to loss of convergent validity. Moreover, GOALS cannot provide procedure-specific feedback that CATs do [2, 3, 6, 33] and may therefore not be useful for in-depth performance assessment. However, the fact that expert GOALS scores showed a strong correlation with expert MIE-CAT scores, suggest that expert GOALS scores may be used in situations where quicker screening of operative skills is required. If expert GOALS scores deviate from desired scores, this screening may be followed by in-depth review using the MIE-CAT, enabling the benefits of the MIE-CAT for detailed feedback, training, and quality improvement. Such a strategy might be a more efficient way to use the current tools that are available.

This study had several limitations. First, the crowd workers’ training included a skill comparison test with dry-lab videos. Ideally, this training would be more extensive [30] and should have included low- and high-performance surgical MIE videos. Unfortunately, in the beginning of this study these examples did not exist and therefore were not available. Regardless, we question how relatively short instructions can be used to effectively score complex surgical videos. Second, the most representative content for video clip performance assessment for MIE remains unclear. Current video clips were selected by the study team based on procedural-phase landmarks (e.g., introducing the stapler for the creation of the gastric tube) to provide comparable video clips. Successfully, a strong correlation between the assessed video clips with GOALS and full-length surgical videos with the MIE-CAT was observed. We expect performance in video clips will be representative to performance in full-length videos, even when components such as a large bleeding are absent. Nonetheless, future research is desired to determine the optimal video clip content and length, so video clips are representative for the complete procedure. Third, with the current study design we could not determine the individual influence of GOALS and video clips on the validity of global performance assessment of MIE by experts. Additional research would be recommended to investigate this for future use.

Once again video assessment has proven to be very labor intensive. Until performance assessments can be automated with artificial intelligence [11], further research could explore the reliability and validity of (para)medical workers, e.g., OR assistants or surgeons in training, for surgical performance assessment of complex laparoscopic procedures. [34] In addition, video clip assessment with GOALS seems to provide a fair reflection of global performance assessment of MIE if executed by experts and could be investigated to advance MIE video assessments. Experts’ GOALS assessments could be implemented in large-scale global performance video clip assessment as a screening or research tool for global performance levels, e.g., nationwide screening for performance of a specific procedural step, such as the creation of the anastomosis. Moreover, combining global performance assessment for initial screening with procedure-specific assessment for specific feedback and research questions could help optimizing essential MIE-CAT steps and minimize assessment time. Subsequently, shorter assessment time would enhance applicability of the MIE-CAT in daily clinical use. At the same time, a valuable database for computer-assisted video assessments containing global and procedure-specific performance assessments would be collected.

## Conclusion

This study showed that global performance assessment of MIE by crowd workers is not useful for assisting expert assessments. MIE might be technically too difficult to assess for laypeople. GOALS, if used by experts, could be considered for large-scale global MIE performance video clip assessments and could be useful to shorten assessment time. However, as GOALS is not comprehensive enough to assess MIE performance in detail, the MIE-CAT can be used for an extensive procedure-specific performance analysis.

### Supplementary Information

Below is the link to the electronic supplementary material.

Supplementary file1 (DOCX 80 kb)
