# Supplementary material for: Crowd-sourced and expert video assessment in minimally invasive esophagectomy
Source: Surg Endosc. 2023 Aug 21;37(10):7819–28. doi: 10.1007/s00464-023-10297-2 (PMC10520122; doi:10.1007/s00464-023-10297-2)
Supplement: Supplementary file 1 — Supplementary file1 (DOCX 80 kb) [file 464_2023_10297_MOESM1_ESM.docx]

**Appendix**

**Appendix A – Overview method video clip selection**

**Appendix B - Hypotheses for convergent validity**

**Appendix C – Overview patient demographics**

**Appendix D – MIE-CAT scores videos**

**Appendix E – Ranking of the videos according to GOALS scores**

**Appendix A - Overview method video clip selection**

*Experience groups*

Videos were divided in 4 groups, based on the experience of the surgical team expressed in conductive case date and the learning curve of 119 cases found by van Workum et al:

- Novice: surgical team having performed 0-25 MIEs.
- Intermediate: surgical team having performed 26-119 MIEs.
- Advanced: surgical team having performed 120-200 MIEs.
- Expert: surgical team having performed > 200 MIEs.

Thresholds for these groups were based on the learning curve to reach an 8% plateau for anastomotic leakage, derived from the same dataset as these videos. First, within the first 25 cases the highest anastomotic leakage was observed (novice). Second, the learning curve of 119 cases to reach the 8% plateau for anastomotic leakage was used (intermediate). Third and fourth, after 200 cases the study team expected the surgical team to have a complete stable MIE procedure (at least ‘assessment’ stage (3) in the IDEAL framework) as described by Stenstra et al.

van Workum et al. "Learning curve and associated morbidity of minimally invasive esophagectomy: a retrospective multicenter study." Annals of surgery 269.1 (2019): 88-94

McCulloch, Peter, et al. "No surgical innovation without evaluation: the IDEAL recommendations." The Lancet 374.9695 (2009): 1105-1112.

Stenstra, Marianne HBC, et al. "Evolution of the surgical technique of minimally invasive Ivor-Lewis esophagectomy: description according to the IDEAL framework." Diseases of the Esophagus 32.3 (2019): doy079.

*Procedural phase-based video clips*

Videos clips were edited so each clip was representative to one of the eight procedural phases of the MIE-CAT. Cut-off points were based on the Delphi study by Eddahchouri et al., who identified 106 essential procedural steps:

| Phase | Description | Start | End |
| --- | --- | --- | --- |
| 1 | Mobilization of the greater curvature | First dissection gastrocolic ligament along greater curvature just cranial of the transverse colon | Last dissection of the greater curvature around the spleen, just before moving on to the crus (+/- 10 min after start) |
| 2 | Mobilization of the lesser curvature | Start of opening of gastro-hepatic ligament onto the stomach | Stomach is completely mobilized onto the diaphragm (+/- 10 min after start) |
| 3 | Dissection of the abdominal lymph nodes | First movement after identification of left gastric artery and vein | Transection of left gastric artery (+/- 10 min after start) |
| 4 | Dissection of the hiatus | First dissection of peritoneum around the distal esophagus in the hiatus | Last dissection of hiatus before moving to creation of the gastric tube (+/- 10 min after start) |
| 5 | Creation of the gastric tube | Insertion of the linear stapler to create the gastric tube | Checking hemostasis along the staple line after firing staples (+/- 10 min after start) |
| 6 | Mobilization of the thoracic esophagus | First dissection in pleura to mobilize the esophagus in the thorax | Dissection of pleura before lymph node dissection (+/- 10 min after start) |
| 7 | Dissection of the thoracic lymph nodes | First dissection in pleura to identify the thoracic lymph nodes | Removing one thoracic lymph node (+/- 10 min after start) |
| 8 | Creation of the intrathoracic anastomosis. | Inserting anvil to create the anastomosis | Anvil has been secured into the esophagus (+/- 10 min after start) |

Eddahchouri Y, van Workum F, van den Wildenberg FJ, van Berge Henegouwen MI, Polat F, van Goor H, Pierie J-PE, Klarenbeek BR, Gisbertz SS, Rosman C (2021) European consensus on essential steps of Minimally Invasive Ivor Lewis and McKeown Esophagectomy through Delphi methodology. Surgical Endoscopy:1-15

**Appendix B – Hypotheses for convergent validity**

| **Correlation with** | **GOALS component scores** | **Expected correlation** | **Explanation** |
| --- | --- | --- | --- |
| Experience of the surgical team | Domain (4)  Phase (1)  Total (1) | 0.3-0.7 (+) | Moderate positive correlation, as experience is expected to be an indicator for global performance of MIE: more experience, increased global performance. Lower correlation would indicate inadequate performance assessment, higher correlation would indicate only correlation with experience while performance is expected to embody more. |
| Clinical parameters blood loss (1) and operative time (2) | Domain (4x2)  Phase (1x2)  Total (1x2) | 0.3-0.7 (-) | Moderate negative correlation, as clinical outcomes are expected to be an indicator for global performance of MIE: improved clinical outcomes, increased global performance. Lower correlation would indicate inadequate performance assessment, higher correlation would indicate only correlation with clinical outcomes while performance is expected to embody more. |
| Procedure-specific performance (MIE-CAT) | Domain vs. MIE-CAT quality component (4)  Phase vs. MIE-CAT phase (1)  Total vs. MIE-CAT total (1) | 0.5-0.8 (+) | Moderately strong positive correlation, as increased global performance is expected to correlate with increased procedure-specific performance. GOALS assessment of MIE should at least correlate moderately with the MIE-CAT assessments. Higher correlations are not expected, as the MIE-CAT is more procedure-specific. |

**Appendix C – Overview patient demographics**

eTable 1: Overview patient demographics of the eight included MIE videos.

|  | **Novice**  **(n = 2)** | **Intermediate**  **(n = 2)** | **Advanced**  **(n = 2)** | **Expert**  **(n = 2)** | **Total**  **(n = 8)** |
| --- | --- | --- | --- | --- | --- |
| **Age, median (IQR), years** | 54.5  (44.3-64.8) | 54.0  (52.5-55.5) | 70.0  (68.5-71.5) | 71.0  (70.5-71.5) | 68.5  (55.5-70.5) |
| **Sex**  ***Male (%)***  ***Female (%)*** | 2 (100)  0 | 1 (50)  1 (50) | 2 (100)  0 | 2 (100)  0 | 7 (87.5)  1 (12.5) |
| **BMI, median (IQR), kg/m** | 28.4  (24.3-32.4) | 23.9  (22.8-25.1) | 32.8  (29.8-35.8) | 21.9  (20.4-23.3) | 25.6  (21.3-29.2) |
| **ASA (%)**  I  II  III  IV | 0  1 (50)  1 (50)  0 | 1 (50)  1 (50)  0  0 | 0  1 (50)  1 (50)  0 | 0  1 (50)  1 (50)  0 | 1 (12.5)  4 (50)  3 (37.5)  0 |
| **Tumor type (%)**   - Adenocarcinoma - SCC - Other - Unknown | 1 (50)  1 (50)  0  0 | 2 (100)  0  0  0 | 2 (100)  0  0  0 | 2 (100)  0  0  0 | 7 (87.5)  1 (12.5)  0  0 |
| **Tumor location (%)**   - Middle - Distal - GE junction | 0  2 (100)  0 | 0  2 (100)  0 | 0  2 (100)  0 | 0  2 (100)  0 | 0  8 (100)  0 |
| **Neoadjuvant therapy (%)**   - CRT - Chemo - None | 2 (100)  0  0 | 2 (100)  0  0 | 1 (50)  1 (50)  0 | 0  2 (100)  0 | 5 (62.5)  3 (37.5)  0 |
| **Conversion (%)**   - Abdomen - Thorax | 0  0 | 0  0 | 0  0 | 0  0 | 0  0 |
| **Blood loss, mean (range), ml** | 275  (100-450) | 0  (0-?*) | 200  (50-350) | 50  (50-50) | 150  (0-450) |
| **Operative time, mean (range), min** | 315  (300-329) | 301  (290-311) | 221  (172-270) | 185  (180-190) | 256  (172-329) |

*Missing

**Appendix D – MIE-CAT scores**

The eight full-length MIE videos had a mean total MIE-CAT score of 96.16 [78.67 – 111.99] and an individual mean phase MIE-CAT score of 12.02 [6.33 – 16.00], eTable2 and eFigure1.

eTable 2: Mean MIE-CAT quality component per video clip, phase (video clip) and total video scores assessed by experts.

| MIE-CAT | Experts mean  [range] |
| --- | --- |
| *Exposure*  *(n = 64)* | 2.88  [1.5-4.0] |
| *Execution*  *(n = 64)* | 2.96  [1.0-4.0] |
| *Adverse events*  *(n = 64)* | 3.23  [2.0-4.0] |
| *End-product quality*  *(n = 64)* | 2.95  [0.83-4.0] |
| *Phase scores*  *(n = 64)* | 12.02  [6.3-16.0] |
| *Total MIE-CAT*  *(n = 8)* | 96.16  [78.67-111.99] |

| eFigure 1: Construct validity of the MIE-CAT scores of the 8 videos. |
| --- |

**Appendix E – Ranking of the videos according to GOALS scores**


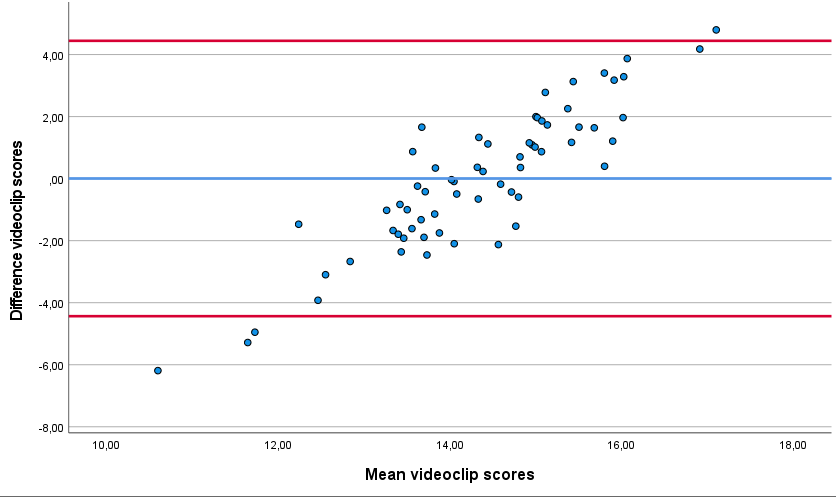


eFigure 2: Bland Altman plot showing the 64 mean GOALS video clip score set against the differences in score between experts and crowd workers on the same video clip. The blue line represents the mean difference (0,06) and the red the 95% limits of agreement (1,96 +/- SD of 2.62).

eTable 3: Total GOALS video scores by experts and crowd workers with their ranking. *Corresponding ranking of videos.

| Vid ID | Expert mean | Raking | Crowd workers mean | Ranking |
| --- | --- | --- | --- | --- |
| *1* | 116.50 | 4 | 111.65 | 7 |
| *2* | 106.75 | 7 | 116.14 | 3 |
| *3* | 128.92 | 1 | 116.29 | 2 |
| *4* | 110.83 | 6 | 114.82 | 5 |
| *5* | 121.17 | 2 | 113.76 | 6 |
| *6* | 99.75 | 8* | 110.83 | 8* |
| *7* | 114.00 | 5 | 117.54 | 1 |
| *8* | 120.08 | 3 | 116.07 | 4 |

eTable 4: Ranking of the 64 video clips according to their GOALS score. *Video in the same cluster ranking of 8 videos (1 to 8, 9 to 16 etc.).

| Video clip ID | Expert GOALS score | Ranking | Crowd GOALS score | Ranking |
| --- | --- | --- | --- | --- |
| 1 | 15 | 25 | 13,67 | 60 |
| 2 | 12,5 | 53 | 14,17 | 40 |
| 3 | 15 | 26 | 13,88 | 53 |
| 4 | 16,5 | 9* | 14,86 | 10* |
| 5 | 14,5 | 28 | 12,84 | 64 |
| 6 | 15,5 | 20 | 14,41 | 26 |
| 7 | 14 | 34 | 14,09 | 47 |
| 8 | 13,5 | 41 | 13,74 | 57 |
| 9 | 11,5 | 58 | 14,17 | 41 |
| 10 | 12,25 | 57 | 14,61 | 21 |
| 11 | 13 | 45 | 14,75 | 13 |
| 12 | 16 | 14* | 14,83 | 11* |
| 13 | 15,5 | 21* | 14,48 | 22* |
| 14 | 14,5 | 29 | 14,93 | 9 |
| 15 | 13,5 | 42 | 13,92 | 52 |
| 16 | 10,5 | 61 | 14,42 | 25 |
| 17 | 12,75 | 50 | 14,36 | 29 |
| 18 | 16 | 15 | 15,6 | 2 |
| 19 | 16 | 16 | 14 | 50 |
| 20 | 17,67 | 4 | 14,38 | 28 |
| 21 | 17 | 7 | 13,87 | 54 |
| 22 | 19 | 2 | 14,82 | 12 |
| 23 | 15,5 | 22* | 14,63 | 20* |
| 24 | 15 | 27 | 14,64 | 18 |
| 25 | 16 | 17 | 14,03 | 48 |
| 26 | 13,83 | 40 | 14,32 | 32 |
| 27 | 14 | 35 | 15,53 | 3 |
| 28 | 12,75 | 51 | 14,64 | 19 |
| 29 | 14,5 | 30 | 14,68 | 15 |
| 30 | 17,5 | 5 | 14,1 | 45 |
| 31 | 9,25 | 62 | 14,2 | 39 |
| 32 | 13 | 46 | 13,83 | 55 |
| 33 | 16,5 | 10 | 13,72 | 58 |
| 34 | 15,17 | 24* | 14,46 | 23* |
| 35 | 13 | 47 | 14 | 51 |
| 36 | 12,5 | 54 | 14,29 | 34 |
| 37 | 12,5 | 55 | 14,42 | 24 |
| 38 | 18 | 3 | 14,13 | 43 |
| 39 | 19,5 | 1 | 14,7 | 14 |
| 40 | 14 | 36 | 14,03 | 49 |
| 41 | 14 | 37 | 13,66 | 61 |
| 42 | 11,5 | 59* | 12,97 | 63* |
| 43 | 7,5 | 64* | 13,69 | 59* |
| 44 | 9 | 63 | 14,28 | 35 |
| 45 | 14,5 | 31 | 15,09 | 6 |
| 46 | 14 | 38 | 13,13 | 62 |
| 47 | 12,75 | 52* | 13,77 | 56* |
| 48 | 16,5 | 11 | 14,24 | 38 |
| 49 | 17,5 | 6 | 14,32 | 31 |
| 50 | 13,5 | 43 | 15,63 | 1 |
| 51 | 11 | 60 | 14,1 | 46 |
| 52 | 12,5 | 56 | 14,96 | 8 |
| 53 | 14 | 39 | 14,66 | 17 |
| 54 | 13 | 48 | 14,32 | 33 |
| 55 | 16 | 18 | 14,27 | 36 |
| 56 | 16,5 | 12 | 15,29 | 4 |
| 57 | 17 | 8* | 15,03 | 7* |
| 58 | 16,33 | 13* | 14,67 | 16* |
| 59 | 14,5 | 32 | 14,27 | 37 |
| 60 | 13,25 | 44 | 14,39 | 27 |
| 61 | 16 | 19 | 14,14 | 42 |
| 62 | 15,5 | 23 | 14,35 | 30 |
| 63 | 13 | 49 | 15,1 | 5 |
| 64 | 14,5 | 33 | 14,13 | 44 |

eTable 5: Ranking of videos according to the domain depth perception. *Corresponding ranking.

| Vid ID | Expert GOALS video clip score, mean (95% CI) | Raking | Crowd workers GOALS video clip score, mean (95% CI) | Ranking |
| --- | --- | --- | --- | --- |
| *1* | 3.75 (3.25-4.25) | 3 | 3.31 (3.12-3.50) | 6 |
| *2* | 3.38 (3.01-3.75) | 7 | 3.504 (3.37-3.64) | 2 |
| *3* | 4.15 (3.70-4.60) | 1 | 3.495 (3.36-3.63) | 3 |
| *4* | 3.53 (2.92-4.15) | 6 | 3.35 (3.25-3.44) | 5 |
| *5* | 3.89 (3.47-3.30) | 2 | 3.2406 (3.04-3.44) | 7 |
| *6* | 3.13 (2.36-3.89) | 8* | 3.2410 (3.04-3.44) | 8* |
| *7* | 3.63 (3.04-4.21) | 5 | 3.59 (3.47-3.70) | 1 |
| *8* | 3.74 (3.35-4.13) | 4 | 3.46 (3.27-3.65) | 4 |

eTable 6: Ranking of videos according to the domain bimanual dexterity. *Corresponding ranking.

| Vid ID | Expert GOALS video clip score, mean (95% CI) | Raking | Crowd workers GOALS video clip score, mean (95% CI) | Ranking |
| --- | --- | --- | --- | --- |
| *1* | 3.35 (3.05-3.95) | 7* | 3.56 (3.39-3.73) | 7* |
| *2* | 3.44 (3.09-3.79) | 6 | 3.61 (3.51-3.70) | 5 |
| *3* | 3.81 (3.22-4.40) | 2 | 3.74 (3.60-3.89) | 1 |
| *4* | 3.51 (2.91-4.12) | 4 | 3.71 (3.53-3.88) | 3 |
| *5* | 3.97 (3.52-4.42) | 1 | 3.66 (3.58-3.73) | 4 |
| *6* | 3.25 (2.80-3.70) | 8* | 3.53 (3.35-3.71) | 8* |
| *7* | 3.50 (2.91-4.09) | 5 | 3.59 (3.44-3.74) | 3 |
| *8* | 3.60 (3.33-3.88) | 3 | 3.72 (3.61-3.84) | 2 |

eTable 7: Ranking of videos according to the domain efficiency. *Corresponding ranking.

| Vid ID | Expert GOALS video clip score, mean (95% CI) | Raking | Crowd workers GOALS video clip score, mean (95% CI) | Ranking |
| --- | --- | --- | --- | --- |
| *1* | 3.44 (3.17-3.71) | 6 | 3.52 (3.35-3.68) | 7 |
| *2* | 3.31 (2.87-3.76) | 7 | 3.65 (3.50-3.79) | 2 |
| *3* | 3.81 (3.22-4.40) | 3 | 3.57 (3.41-3.72) | 6 |
| *4* | 3.56 (3.01-4.12) | 4 | 3.65 (3.45-3.85) | 3 |
| *5* | 3.94 (3.33-4.55) | 1 | 3.64 (3.48-3.80) | 4 |
| *6* | 2.88 (2.05-3.71) | 8* | 3.44 (3.24-3.64) | 8* |
| *7* | 3.53 (3.11-3.96) | 5 | 3.76 (3.59-3.93) | 1 |
| *8* | 3.92 (3.36-4.47) | 2 | 3.59 (3.50-3.68) | 5 |

eTable 8: Ranking of videos according to the domain tissue handling. *Corresponding ranking.

| Vid ID | Expert GOALS video clip score, mean (95% CI) | Raking | Crowd workers GOALS video clip score, mean (95% CI) | Ranking |
| --- | --- | --- | --- | --- |
| *1* | 3.88 (3.58-4.17) | 2 | 3.57 (3.43-3.72) | 8 |
| *2* | 3.22 (2.64-3.80) | 8 | 3.76 (3.65-3.86) | 2 |
| *3* | 4.34 (4.01-4.68) | 1 | 3.73 (5.58-3.89) | 4 |
| *4* | 3.25 (2.86-3.64) | 6 | 3.72 (3.51-3.92) | 5 |
| *5* | 3.35 (2.50-4.21) | 5 | 3.69 (3.55-3.82) | 6 |
| *6* | 3.22 (3.59-3.85) | 7* | 3.65 (3.43-3.86) | 7* |
| *7* | 3.59 (3.05-1.14) | 4 | 3.76 (3.58-3.94) | 1 |
| *8* | 3.75 (3.36-4.14) | 3 | 3.74 (3.62-3.87) | 3 |
